# *Staphylococcus epidermidis* Isolated in 1965 Are More Susceptible to Triclosan than Current Isolates

**DOI:** 10.1371/journal.pone.0062197

**Published:** 2013-04-16

**Authors:** Sissel Skovgaard, Lene Nørby Nielsen, Marianne Halberg Larsen, Robert Leo Skov, Hanne Ingmer, Henrik Westh

**Affiliations:** 1 Department of Clinical Microbiology, Hvidovre Hospital, Copenhagen, Denmark; 2 Department of Veterinary Disease Biology, Faculty of Health and Medical Sciences, University of Copenhagen, Copenhagen, Denmark; 3 Department of Microbiological Surveillance and Research, Statens Serum Institut, Copenhagen, Denmark; 4 Department of Clinical Medicine, Faculty of Health and Medical Sciences, University of Copenhagen, Copenhagen, Denmark; University of Birmingham, United Kingdom

## Abstract

Since its introduction to the market in the 1970s, the synthetic biocide triclosan has had widespread use in household and medical products. Although decreased triclosan susceptibility has been observed for several bacterial species, when exposed under laboratory settings, no *in vivo* studies have associated triclosan use with decreased triclosan susceptibility or cross-resistance to antibiotics. One major challenge of such studies is the lack of strains that with certainty have not been exposed to triclosan. Here we have overcome this challenge by comparing current isolates of the human opportunistic pathogen *Staphylococcus epidermidis* with isolates collected in the 1960s prior to introduction of triclosan to the market. Of 64 current *S. epidermidis* isolates 12.5% were found to have tolerance towards triclosan defined as MIC≥0.25 mg/l compared to none of 34 isolates obtained in the 1960s. When passaged in the laboratory in the presence of triclosan, old and current susceptible isolates could be adapted to the same triclosan MIC level as found in current tolerant isolates. DNA sequence analysis revealed that laboratory-adapted strains carried mutations in *fabI* encoding the enoyl-acyl carrier protein reductase isoform, FabI, that is the target of triclosan, and the expression of *fabI* was also increased. However, the majority of the tolerant current isolates carried no mutations in *fabI* or the putative promoter region. Thus, this study indicates that the widespread use of triclosan has resulted in the occurrence of *S. epidermidis* with tolerance towards triclosan and that the adaptation involves FabI as well as other factors. We suggest increased caution in the general application of triclosan as triclosan has not shown efficacy in reducing infections and is toxic to aquatic organisms.

## Introduction

Triclosan is a synthetic broad spectrum biocide that was introduced to the market in the early 1970s [1]. In low concentrations triclosan inhibits the growth of many bacteria and higher concentrations can be bactericidal [2], [3]. It is now widely used as an antiseptic, disinfectant and preservative in clinical settings and in various consumer products including cosmetics, plastic materials, toys and textiles [4]. Environmental exposure, toxicity and mechanisms of action have recently been reviewed by Dann et Hontela [5]. Triclosan is excreted in the urine and has been found in human urine (2.4–3790 µg/l), plasma (0.01–38 µg/l) and breast milk (0.018–0.95 µg/l). The extensive use in human products has led to accumulation in the environment with concentrations of 20–133,000 µg/kg dry-weight in biosolids from wastewater treatment plants. Very low concentrations, in the ng/l range, have been found in lakes, rivers, seawater and drinking water. Toxicological studies indicate that triclosan is not toxic for mammals, however, triclosan has an estimated bioaccumulation factor of more than 1000 in algae and can be highly toxic to green algae. [4]–[6]

It has been wondered whether the extensive use of triclosan could select for antimicrobial resistance. For staphylococci only low-level triclosan resistance, tolerance, has been detected and the few exposure studies that have been conducted, have not shown any dependency between triclosan usage and increased tolerance towards triclosan or co/cross-resistance to antibiotics [7]–[10]. A confounder for exposure studies is the omnipresence of triclosan in the environment, which affects both the user groups and the controls [7]. *In vitro* studies have shown that bacteria can be stably adapted to increasing concentrations of triclosan and in some cases develop cross-resistance towards antibiotics. This has mostly been found for Gram-negative rods, caused by multidrug efflux pumps [11]–[14].

*Staphylococcus epidermidis* is an abundant human skin commensal as well as an important opportunistic pathogen responsible for a significant number of severe foreign body related infections. *S. epidermidis* isolated from blood cultures span the clinical spectrum from skin contaminants to severe infections [15]. *S. epidermidis* are exposed to triclosan through personal care products and the incidence of triclosan tolerance have been found to be higher in clinical *S. epidermidis* than in clinical *Staphylococcus aureus* isolates and it is speculated if the mechanisms are similar [16]. In *S. aureus* the mechanism for tolerance has been recognized as mutations in or increased expression of the gene *fabI*. The FabI enzyme, enoyl-acyl carrier protein reductase, catalyses the final step in bacterial type II fatty acid biosynthesis. Triclosan functions as a slow binding inhibitor that inactivates the enzyme through the formation of a stable, non-covalent, FabI-NAD+−triclosan ternary complex. [17]

In this study we describe, for the first time, the susceptibility towards triclosan in *S. epidermidis* that have never been exposed to triclosan, namely *S. epidermidis* isolated from blood in 1965–66, well before the introduction of triclosan to the market. We compare these old isolates with current isolates of *S. epidermidis*. The old *S. epidermidis* were exposed to triclosan, leading to development of tolerance to triclosan. The *fabI* gene and its expression was characterized in laboratory developed triclosan tolerant *S. epidermidis* and current triclosan tolerant *S. epidermidis*.

## Methods

### Ethics

Scientific ethical committee approval is not required for this project. We worked with bacteria that were already isolated from blood cultures as part of the standard operating procedures with samples referred to the Department of Clinical Microbiology at Hvidovre Hospital, Denmark. No material relating to humans was used or stored in this experiment and all person related data were blinded for the researchers. This studies results or the researchers had no influence on any aspects concerning patient care.

### Bacterial isolates

A collection of coagulase negative staphylococci isolated from blood cultures of Danish patients during 1965 and 66 has been stored in agar sticks at Statens Serum Institut, Copenhagen, Denmark and have stayed untouched since then. From 149 of those old agar sticks isolates could be cultured from 51, and 34 of these isolates were identified as *S. epidermidis.* These 34 isolates were used as pre-triclosan era controls in relation to triclosan exposure. As comparable isolates, which have been exposed to “the uncontrolled” modern usage of triclosan, 64 *S. epidermidis* isolates were collected from blood cultures of patients hospitalized in Copenhagen, Denmark, 2010–11. We collected these 64 isolates consecutively, until we had 40 methicillin resistant *S. epidermidis*. After that we continued to collect methicillin susceptible isolates for a total of 24 to increase the diversity of the collection.

All isolates were species-identified by MALDI-TOF MS (Bruker Daltonics), *S. epidermidis* ATCC 12228 (NCBI Reference Sequence: NC_004461.1) was included in the susceptibility tests as control. Isolates were onward stored at −80°C. Tryptic soy broth/agar (TSB/TSA) (Oxoid, Roskilde, Denmark) was used as growth medium unless otherwise stated. All incubations were performed at 37°C atmospheric air.

### Antimicrobial and biocide susceptibility testing

The MIC of triclosan was determined by following the recommendations of the British Society of Antimicrobial Chemotherapy using broth micro dilutions [18]. A stock solution of triclosan (Irgasan, SIGMA-ALDRICH®, Germany) of 1 mg/ml dissolved in 96% ethanol (KEMETYL A/S, Denmark) was prepared in advance and a doubling dilution range from 0.0156–16 mg/l triclosan in Mueller Hinton bouillon (MHB) (Oxoid, Roskilde, Denmark) was made for each experiment. 100 µl of the triclosan dilution+100 µl of an overnight bacterial suspension adjusted to 10^6^ CFU/ml was mixed in each well. The MIC was determined as the lowest concentration that inhibited visible growth after 24 hours. Positive (bacterial suspension+MHB) and negative (MHB and triclosan dilutions without bacterial suspension) controls were included in each measurement. Bacterial growth in the highest used dilution of 96% ethanol (0.77%) was also tested and did not differ visually from growth in MHB alone. 10 µl from each well (0.0625 mg/l and above) was, after 24 hours incubation spotted on to Muller-Hinton agar (MHA) plates containing no triclosan. The MBC was read as the lowest concentration with no growth after 48 hours. All MIC experiments were repeated on two separate occasions each with two technical replicates and MIC is given as the mean. The MBC was repeated for all isolates with a high MBC defined as 8 mg/l and for one third of the rest (below 8 mg/l). All of the repeat MBCs stayed at 8 mg/l or below 8 mg/l, respectively. Antibiotic susceptibility was determined using the disc diffusion method (Oxoid, Roskilde, Denmark) following the EUCAST guidelines and breakpoints [19], [20].

### Adaptation experiments

Six *S. epidermidis* isolates were adapted to triclosan using the gradient plate approach [21]. Initial gradients consisted of zero mg/l at one end of the plate up to 0.025 mg/l or 4 mg/l at the other end depending on the initial MIC of each isolate. After 48 h of incubation, colonies from the leading edge of growth were passed to a new gradient plate with the same concentrations until growth occurred across the entire plate. This process was repeated using doubling increases in triclosan gradients until no further adaptation was possible. The current *S. epidermidis* isolates, with a high triclosan MIC, were passed repeatedly on plates going up to 4 mg/l as many times as the other isolates were passed. Each isolate was passed in duplicate and with a control passed along on TSA plates without triclosan. Colonies from the leading edges from the final gradient plates as well as controls were frozen at −80°C. Furthermore, colonies from the leading edges of each of the adapted isolates were passed on TSA without triclosan for 5 days and then frozen. Adapted isolates, controls and adapted isolates passed for 5 days without triclosan were subjected to triclosan susceptibility testing and antibiotic susceptibility testing.

### *FabI* sequencing and MLST

Template DNA was prepared by suspending colonies from an overnight culture in Milli-Q water, heating at 100°C for 10 minutes, centrifugation at 8000 rpm for to minutes and then the supernatant was used immediately or frozen at −20°C.

The *fabI* gene including the putative promoter region in *S. epidermidis* was amplified using the primers 5′-GGTGTTGTTGAAGATCAAATATAC-3′ and 5′-GTCCTCTTATTAAACTCCG-3′.

Multilocus sequence typing (MLST) was conducted following the mlst.net guidelines and the same thermocycler program was used for *fabI* as for the MLST reactions [22]. PCR products were purified using 1 µl exonuclease1 and 2 µl alkaline phosphatase (Fermentas, Roskilde, Denmark) for 10 µl PCR product, activated at 37° C for 15 minutes and terminated at 85°C for 15 minutes. The purified products were sequenced at both strands using the same primer set as amplification and Macrogenservice (Macrogen Europe, Netherlands). The results were analyzed using CLC Main Workbench 6.2.

### RNA extraction and northern hybridization

Cells of *S. epidermidis* were grown to mid logarithmic growth phase (OD600 = 0.6–0.8) in MH broth and samples were immediately cooled in ice-water bath. The bacterial cells were lysed using the Fast Prep FP120 instrument (BIO101, ThermoSavent) for 45 s at speed 6.0. Total RNA was extracted from the cells using the RNeasy mini kit (Qiagen, Denmark) according to the manufacturer's directions. Analysis of transcripts was done as previously described [23]. Hybridization probes were generated by PCR from chromosomal DNA of *S. aureus* 8325–4 using specific primers for the *fabI* gene (fab2F: atgttaaatcttgaaaacaaaac and fab2R: ttatttaattgcgtggaatccgc), (TAG Copenhagen A/S, Denmark). RNA extracted from at least two independent experiments was analyzed.

### Statistics

Based on a pilot study (data not shown) showing that none of 22 old *S. epidermidis* isolates were triclosan tolerant while 15% of current isolates were, a power analyses was performed using G*Power 3.1.5 (freely distributed at http://www.psycho.uni-duesseldorf.de/abteilungen/aap/gpower3/download-and-register). Given a power of 0.80 and α = 0.05 a required sample size of minimum 32 old isolates and 59 current isolates was calculated.

Categorical variables were compared using Fisher's exact test.

## Results

### Triclosan susceptibility in old and in current *S. epidermidis* isolates

The 34 old *S. epidermidis* isolates were all methicillin susceptible. The results of the triclosan MIC/MBC determinations are summarized in Table 1. Old and current *S. epidermidis* isolates had the same MIC50 (MIC required to inhibit the growth of 50% of isolates) of 0.0625 mg/l and for the old isolates the MIC90 (MIC required to inhibit the growth of 90% of isolates) was also 0.0625 mg/l. This was in contrast to the current isolates that had a MIC90 that was 8 fold higher than the MIC50. The highest MIC value among the current isolates was 4 mg/l; 32-fold higher than the highest MIC value of 0.125 mg/l, among the old isolates. The same pattern was true for the MBC values though on a narrower scale.

**Table 1:** Susceptibility to triclosan among *S. epidermidis* isolates from 1965–66 (old) and from 2010–11 (current) as determined by their minimum inhibitory concentration (MIC) and minimum bactericidal concentration (MBC).

	Number	MIC50	MIC90	MIC range	MBC50	MBC90	MBC range
**Old**	34	0.0625	0.0625	≤0.0156–0.125	1	2	≤0.0625–4
**Current**	64	0.0625	0.5	≤0.0156–4	2	8	≤0.0625–8

MIC/MBC50 = Minimum Inhibitory/Bactericidal Concentration required to inhibit the growth/kill of 50% of isolates. MIC/MBC90 = Minimum Inhibitory/Bactericidal Concentration required to inhibit the growth/kill of 90% of isolates

**Figure 1:**
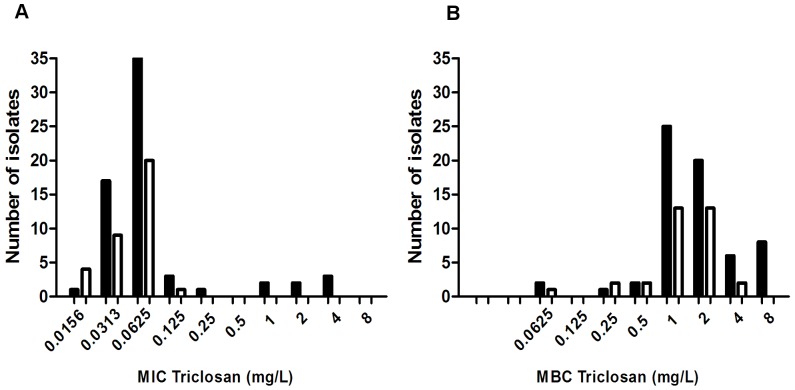
Triclosan MIC and MBC distributions for *S. epidermidis* isolates from 1965-66 and from 2010-11. A: Triclosan MIC (Mininmum inhibitory concentration) distributions. B: Triclosan MBC (Mininmum bactericidal concentration) distributions. Black bars = *S. epidermidis* blood isolates from 2010-11. White bars = *S. epidermidis* blood isolates from 1965-66. The lowest dilutions represent a MIC of≤0.0156 mg/l and a MBC of≤0.0652 mg/l. Eight isolates in the current group have a MIC ≥0.25 mg/l and the same eight isolates are the ones with a MBC = 8 mg/l.

The distributions of the MIC and MBC values are shown in Figure 1. A group of 8 isolates were identified amongst the current isolates having a MIC≥0.25 mg/l and a MBC of 8 mg/l not seen among the old isolates. This suggests a wild-type population cut-off MIC of 0.25 mg/l and MBC of 8 mg/l. By this definition the isolates fell in two natural groups (Figure 1, Table 2) and based on their MIC values they could be divided into triclosan susceptible isolates (MIC<0.25 mg/l) and triclosan tolerant (MIC≥0.25 mg/l). The current *S. epidermidis* isolates had significantly more triclosan tolerant isolates compared to the group of old isolates; 12.5% versus 0%. This was even more evident when comparing current methicillin resistant *S. epidermidis* isolates to the old isolates, but triclosan tolerance was also seen in the current methicillin susceptible *S. epidermidis* isolates (Table 2).

**Table 2:** Distribution of triclosan tolerance among *S. epidermidis* isolates from 1965-66 (old) and 2010-11 (current).

	N	MIC≥0.25 mg/l	Fishers exact test^a^
**Old**	34	0 (0%)	
**Current**	64	8 (12.5%)	p≤0.048
**Current, cefoxitin S**	24	2 (8.3%)	Not significant
**Current, cefoxitin R**	40	6 (15%)	p≤0.028

MIC <0.25 mg/l is defined as susceptible and MIC ≥0.25 mg/l is defined as tolerant.

a Old isolates versus current isolates.

### Triclosan tolerant isolates are not clonal

MLST results (supplementary material, Table S1) revealed that the triclosan tolerant isolates were not due to the expansion of a clone. Out of 15 current isolates (seven triclosan susceptible and the eight triclosan tolerant) nine different ST types were found with six different ST types in the group of triclosan tolerant isolates and three ST types were represented in both the susceptible and the tolerant isolates.

### Old *S. epidermidis* isolates could be adapted to triclosan tolerance

Two old (65–13, 66–1) and two current (BD-62, Van-1) isolates, all triclosan susceptible, and two current isolates with a high triclosan MIC (BD-12, BD-24) were attempted adapted to triclosan. The four triclosan susceptible isolates all acquired stable tolerance to triclosan. Three isolates maintained a stabile triclosan MIC of 4 mg/l, while one old isolate peaked at MIC of 2 mg/l and following 5 passages without triclosan dropped to 0.375 mg/l, while maintaining a MBC of 8 mg/l. It was not possible to increase the MIC of the two isolates that were already triclosan tolerant with an MIC of 4 mg/l. Results are presented in Table 3 left side columns.

**Table 3:** Triclosan and antibiotic susceptibility of parental strains from 1965-66 (65–13 and 66–01) and from 2010–11 (BD-62, Van-1, BD-12 and BD-24) and their triclosan laboratory exposed descendents.

Isolate ID (ST)	MIC_0	MBC_0	MIC_5	MBC_5	PEN	FOX	FA	GEN	ERY	CLIN	RIF	LIN	NOF
65-13 (410)	0.0625	0.25			R	S	S	S	S	S	S	S	S
65-13a	2	8	0.25	8	R	S	S	S	S	S	S	S	S
65-13b	2	8			R	S	S	S	S	S	S	S	S
65-13Ka	0.0313	0.125			R	S	S	S	S	S	S	S	S
65-13Kb	0.0313	0.25											
66-1 (190)	0.125	4			R	S	S	S	S	S	S	S	S
66-1a	4	8	4	8	R	S	S	S	S	S	S	S	S
66-1b	4	8			R	S	S	S	S	S	S	S	S
66-1Ka	0.0625	4			R	S	S	S	S	S	S	S	S
66-1Kb	0.0625	4											
BD-62 (327)	0.0313	1			S	S	S	S	S	S	S	S	S
BD-62a	4	4	4	4	S	S	S	S	S	S	S	S	S
BD-62b	4	4			S	S	S	S	S	S	S	S	S
BD-62Ka	0.0313	1			S	S	S	S	S	S	S	S	S
BD-62Kb	0.0313	1											
Van-1 (2)	0.0625	1			R	R	R	R	R	R	S	S	R
Van-1a	4	8	4	8	R	R	R	R	S	S	S	S	R
Van-1b	4	8			R	R	R	R	S	S	S	S	R
Van-1Ka	0.0313				R	R	R	R	S	S	S	S	R
Van-1Kb	0.0313	2											
BD-12 (88)	2	8			R	R	S	S	S	S	S	S	S
BD-12a	4	8	4	8	R	R	S	S	S	S	S	S	S
BD-12b	4	8			R	R	S	S	S	S	S	S	S
BD-12Ka	4	8			R	R	R	S	R	S	S	S	S
BD-12Kb	4	8											
BD-24 (ND)	4	8			R	R	R	S	R	S	S	S	S
BD-24a	4	8	4	8	R	R	R	S	R	S	S	S	S
BD-24b	4	8			R	R	R	S	R	S	S	S	S
BD-24Ka	4	8			R	R	R	S	R	S	S	S	S
BD-24Kb	4	8											

Adapted strains are named with the parent name and the suffix a and b. The control strains passed along without triclosan have the suffix Ka and Kb. MIC_0/MBC_0, measured after ended adaptation. MIC_5/MBC_5, measured after 5 passages on triclosan free media. PEN = penicillin, FOX = cefoxitin, FA = fucidic acid, GEN = gentamicin, ERY = erythromycin, CLIN = clindamycin, RIF = rifampicin, LIN = linezolid, NOF = norfloxacin. ST = Sequence Type, determined by MLST.

### No antibiotic cross-resistance in *S. epidermidis*

There was no significant association between antibiotic resistance and triclosan tolerance in the current isolates (supporting information, Table S2). Furthermore, none of the isolates, old or current, that were adapted to triclosan tolerance developed any resistance towards the tested antibiotics. Indeed one of the adapted isolates, Van-1a and b (see Table 3 for nomenclature), changed from being resistant to become susceptible towards erythromycin and clindamycin but that was also seen in the control strain, Van-1Ka, only passed without triclosan in the media and with no change in triclosan MIC.

### FabI and triclosan susceptibility in *S. epidermidis*

The *fabI* gene of *S. epidermidis* has 82–84% nucleotide similarity to the *fabI* gene of *S. aureus* when blasting published sequences at NCBI. Five old and seven current triclosan susceptible as well as the eight current triclosan tolerant and the six triclosan adapted *S. epidermidis* isolates were *fabI* sequenced (Table 4). We did not get a PCR product from one old (as well as its triclosan adapted isogenic descendent) and two current susceptible isolates and they are excluded from interpretation. The current triclosan susceptible isolates all had the same *fabI* amino acid sequence, identical to the sequence predicted for the *fabI* of the triclosan susceptible *S. epidermidis* ATCC12228 (MIC 0.0625 mg/l and MBC 2 mg/l in our assay). Five of the eight clinical *S. epidermidis* isolates with tolerance towards triclosan also had identical *fabI* amino acid sequence to ATCC 12228 and none of these isolates had mutations in the putative promoter region of *fabI*. The three remaining tolerant *S. epidermidis* isolates had between one and three non synonymous mutations causing changes in the predicted amino acid sequence: Isolate BD-18 had a substitution of a phenylalanine for a valine, at position 204 (F204V), isolate BD-38 had a A198G substitution. Mutations at those two positions have previously been described in *fabI* of *S. aureus* with triclosan tolerance [24]–[27]. BD-24 had three amino acid substitutions a H60Q, a H61Y and a S78A as well as several nucleotide changes in the putative promoter region. None of these substitutions have been described previously and interestingly H61Y and S78A were also found in two old, triclosan susceptible, *S. epidermidis* isolates (65–02, 65–12) while the other two old isolates from which we obtained a *fabI* sequence were identical to the ATCC 12228 *fabI*.

**Table 4:** Analysis of mutations in the *fabI* gene of *S. epidermidis* and the upstream promoter region.

Isolate (year of isolation)	ST	TriclosanMIC mg/l	*fabI* non synonymous mutations	Up-stream (putative promoter region) mutations in relation to current susceptible isolates
65-02 (1965)	414	<0.25	H61Y, S78A, G85S	
65-06 (1965)	86	<0.25	( = ATCC 12228)	
65-12 (1965)	409	<0.25	H61Y, S78A	
65-13 (1965)	410	<0.25	no data	no data
66-01 (1966)	190	<0.25	( = ATCC 12228)	
BD-09 (2010)	5	<0.25	( = ATCC 12228)	Current susceptible
BD-10 (2010)	32	<0.25	( = ATCC 12228)	Current susceptible
BD-26 (2011)	ND	<0.25	no data	no data
BD-44 (2011)	5	<0.25	( = ATCC 12228)	Current susceptible
BD-50 (2011)	73	<0.25	no data	no data
BD-62 (2011)	327	<0.25	( = ATCC 12228)	Current susceptible
Van-1 (2011)	2	<0.25	( = ATCC 12228)	Current susceptible
BD-06 (2010)	88	≥0.25	( = ATCC 12228)	( = current susceptible)
BD-12 (2011)	88	≥0.25	( = ATCC 12228)	( = current susceptible)
BD-18 (2011)	73	≥0.25	F204V	Two NT changes
BD-19 (2011)	88	≥0.25	( = ATCC 12228)	( = current susceptible)
BD-24 (2011)	ND	≥0.25	H60Q, H61Y, S78A	Many NT changes
BD-32 (2011)	2	≥0.25	( = ATCC 12228)	( = current susceptible)
BD-38 (2011)	New	≥0.25	A198G	( = current susceptible)
BD-53 (2011)	5	≥0.25	( = ATCC 12228)	( = current susceptible)
65-13a*		≥0.25	no data	no data
66-01a*		≥0.25	A95V	One NT change compared to 66-01
BD-62a*		≥0.25	A95V	Deletion in −69 to −77 compared to the current susceptible
Van-1a*		≥0.25	A95V	( = current susceptible)
BD-12a*		≥0.25	( = ATCC 12228)	( = current susceptible)
BD-24a*		≥0.25	( = BD-24)	( = BD-24)

The suffix a* indicates adapted strains that have been passed for 5 days on triclosan free media. The *fabI* primers did not work on three isolates (65-13, BD-26 and BD-50). *S. epidermidis* ATCC 12228 is published on NCBI with its full sequence. It has a triclosan MIC <0.25 mg/l and its *fabI* gene is identical to all the current triclosan susceptible isolates we have sequenced. The putative promoter region of ATCC 12228 is different from the current triclosan susceptible isolates we have sequenced but those are all identical. ST, Sequence type determined by MLST. ND = not determined. Abbreviations for amino acids: H = histidine, Y = tyrosine, S = serine, A = alanine, G = glycine, F = phenylalanine, V = valine, Q = glutamine.

Three of the triclosan-adapted isolates, one old and two current, developed an identical mutation that predicted a substitution in amino acid residue 95 from alanine to valine (A95V). This change was not found in any of the current triclosan tolerant *S. epidermidis* isolates, but it has also been identified as the most frequent mutation in *in vitro* selected *S. aureus* mutants with triclosan tolerance [26], [27]. The two triclosan tolerant isolates (that were not possible to adapt further to triclosan in measure of MIC/MBC) did also not develop further mutations in *fabI* following the adaptation experiments with the BD-12 descendent, BD-12a*, keeping the *fabI* sequence identical to ATCC12228, and BD-24a* just keeping the mutations from its parent BD-24 (Table 4).

**Figure 2:**
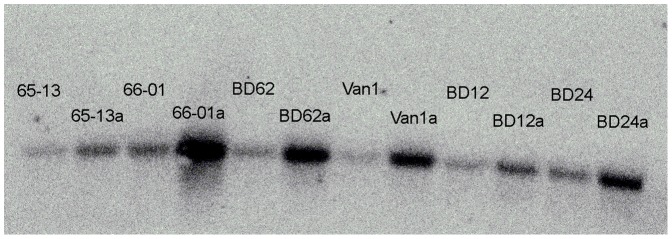
Northern blot analysis of *fabI* transcripts in parental strains and their triclosan adapted descendant. Parental strains from 1965-66 (65-13 and 66-01) and from 2010-11 (BD-62, Van-1, BD-12 and BD-24) and the descendant strains isolated after triclosan adaptation. Adapted strains are named with the parent name and the suffix a.

The mRNA expression of *fabI* of the six parent and six descendant isolates from the adaptation experiment was measured (Figure 2). It was thereby visualized that all the triclosan-exposed descendants had a variable increase in *fabI* expression compared to their own parental isolate including the already tolerant isolates that did not change their MIC/MBC further. When comparing the different isolates, it is seen that the parent isolate BD-12 that had a high MIC/MBC (4 mg/l/8 mg/l) and no mutations in *fabI* or the putative promoter region also had a relatively low mRNA expression of *fabI*.

## Discussion

This study indicates that wild-type *S. epidermidis* has changed their population structure to adapt to the widespread use of triclosan. We now have a triclosan tolerant subpopulation of *S. epidermidis*, so far identified in Denmark and USA [7], [16]. While no Danish isolates from 1965-66 where tolerant, 12.5% of the current isolates studied have decreased triclosan susceptibility with MIC values that were up to 32 fold higher, than the highest value found in the old isolates. We suggest that this change is caused by the general large-scale use of triclosan in society and to a lesser degree in the healthcare system.

We could reproduce this evolution through laboratory experiments exposing triclosan susceptible isolates to increasing concentrations of triclosan. Old, current, antibiotic susceptible, multiresistant and isolates with different ST types could be mutated to increased triclosan MICs. Interestingly it was not possible to adapt *S. epidermidis* isolates to a higher MIC than 4 mg/l and a MBC of 8 mg/l that correlated with the maximum values found in the clinical isolates. A number of studies have examined triclosan tolerance and its mechanisms in *S. aureus* [7], [16], [17], [24]–[26], [28]–[32]. Only two other studies have reported the distribution of triclosan MICs for *S. epidermidis* [7], [16]. In the first study, households were randomized to using or not using liquid soaps containing 0.2% triclosan (2000 mg/l). After one year triclosan MIC values remained in the range ≤0.0312–4 mg/l with no decrease in triclosan susceptibility. The other study investigated clinical *S. epidermidis* collected between 2001 and 2002 from all over USA [16]. They found a triclosan MIC range of ≤0.03–8 mg/l. This is similar to what we have found in the current *S. epidermidis* isolates, and we believe that the prior use of triclosan-containing products may have influenced the overall susceptibility which also may explain why intervention studies have not shown increased MIC levels [7]–[10].

Schmid and Kaplan found that clinical *S. epidermidis* isolates have a higher prevalence of reduced triclosan susceptibility (22%>0.5 mg/l) than clinical *S. aureus* isolates have (5%>0.5 mg/l) and that this was associated with the methicillin resistant (32%) versus the methicillin susceptible phenotype (12%) [16]. We found a non-significant higher prevalence of triclosan tolerant, methicillin resistant *S. epidermidis* isolates (15%) compared to methicillin susceptible *S. epidermidis* isolates (8.3%) (Table 2). Schmidt et al speculated whether the higher incidence of triclosan tolerance in *S. epidermidis* compared to *S. aureus* could be caused by the more frequent contact of *S. epidermidis* with triclosan -containing products or due to an alternative mechanism in *S. epidermidis* than in *S. aureus*.

Different mechanisms of decreased susceptibility towards triclosan have been described for different bacterial species. Moderation of outer membrane permeability barriers and upregulation and adaption of pre-existing effux pumps, is seen in Gram-negative rods for example *P. aeruginosa* and *E. coli* [33]–[35]. In Gram-negative rods also increased expression of the target, the FabI enoyl-ACP reductase, is seen in e.g. *E. coli* and *Salmonella* and mutations in the *fabI gene*, in *E.coli*, *Salmonella*, *P. aeruginosa* and *Mycobacterium* have been described [33]. Some bacterial species harbor an alternative to the FabI enoyl-ACP reductase, e.g. FabK, FabV and FabL, that are not or less affected by triclosan. These bacteria are intrinsically less susceptible to triclosan [33], [36]–[38]. *Streptococcus pneumoniae*, *Enterococcus faecalis*, *Vibrio cholerae, Bacillus subtilis*, *E. coli* and *P. aeruginosa* are examples [36], [37], [39]. Production of triclosan degradative enzymes, has been identified in soil isolates of the bacteria *Pseudomonas putida* and *Alcaligenes xylosoxidans* with intrinsicly reduced susceptibility towards triclosan [40].

In staphylococci, efflux pumps have not been identified as the cause for decreased susceptible to triclosan and only increased expression of the target FabI, and mutations in the *fabI* gene, has been identified. It has been shown that when wild-type *fabI* is introduced to *S. aureus* on a multicopy plasmid the MIC is raised 30 fold from 0.06 mg/l to 2 mg/l [30]. In clinical *S. aureus* isolates, it has been found that increased MIC levels towards triclosan is correlated with both increased expression of *fabI* as well as a single-point mutations in *fabI* [24].

By DNA sequencing of *S. epidermidis fabI* and the putative promoter region we found that mutations in *fabI* are partially involved in the development of triclosan tolerance in this organism, as has been found for *S. aureus*. However, still undefined mechanisms seem to be involved as maximum levels of triclosan tolerance was seen in isolates without mutations in or over expression of *fabI*. Ciusa, Furi and Knight et al [26] recently showed that an additional *fabI* allele most likely achieved by horizontal transfer from *S. haemolyticus* could explain reduced triclosan susceptibility of clinical *S. aureus* isolates with a wild-type *fabI* allele. The homology of the nucleotide sequence between the *fabI* alleles of Staphylococci is in the order of 82–84% (published sequences at NCBI) and we expect that our hybridization probe used in the mRNA expression assay would bind equally well to the *fabI* alleles of *S. epidermidis* and eg *S. haemolyticus.* Therefore yet other mechanisms seem to be involved.

One isolate had a predicted change at amino acid residue 204. An alteration of *fabI* in this position is well known from clinical as well as *in vitro* triclosan selected mutants of *S. aureus* with triclosan tolerance [24], [26]. The mutation has been shown, in *S. aureus fabI*, to correlate with elevated triclosan MIC through the formation of a stable triclosan −NAD+−FabI complex not seen in the wild-type *fabI* [24]. The amino acid residue at position 95 has, just as that at position 204, been thought to lie in the cofactor binding region of *S. aureus fabI* [27]. It is interesting though that the most frequently *in vitro* selected mutation, A95V, that is also identified as the mutation with the greatest impact on inhibition of *S. aureus fabI* [27] has never, as far as we are aware, been detected in clinical staphylococci isolates. An explanation could be that this mutation has a relatively larger fitness cost than the other mutations.

As other *in situ* studies [7]–[10] we did not find a significant correlation between triclosan tolerant isolates and antibiotic resistance in staphylococci. Only the previous discussed study [16] has found such an association, where triclosan tolerance was associated to methicillin resistance in *S. epidermidis*. Importantly we did not see increased antibiotic resistance in our laboratory adapted isolates either. It seems at least that in *S. epidermidis* there is no cross-resistance to antibiotics but the possibility of co-selection *in vivo* with for example methicillin and maybe other, not yet identified traits is still not fully elucidated. We believe, based on others and our findings, that the targeted use of triclosan is safe in regard to development of antibiotic cross-resistance and in working concentrations should be effective against *S. epidermidis*. A claim has been made for using triclosan in medical devices such as sutures with the aim to lower rates of nosocomial infections. Recently a multicenter, prospective, double-blinded, parallel group study was conducted in Finland [41] comparing triclosan-coated suture material with noncoated sutures in the control group. Among the 276 patients undergoing lower limb revascularization surgery, similar surgical wound infection rates of 21.9 to 22.3% were found.

The widespread (and often unregulated) use of triclosan in textiles, chopping boards and in antibacterial soaps in the domestic setting might need to be reconsidered. Aiello et al. [42] reviewed the literature on commonly used soaps containing triclosan in the community setting. In working concentrations 0.1%–0.45% wt/vol (1000–4500 mg/l) triclosan containing soaps were no more effective than plain soap in preventing infectious illness symptoms and reducing bacterial levels on the hands. Finally, taking the environmental concerns about aquatic organisms and toxic by-products into consideration together with the still not fully understood consequences of an increased tolerance in some bacterial species, we think the use of triclosan should be more regulated and restricted for purposes that have been proven beneficial.

## Supporting Information

Table S1 **Multilocus sequence typing (MLST) was performed for 15** ***S. epidermidis*** **isolated from blood in 2010-11, 7 triclosan susceptible (MIC<0.25** **mg/l) and 8 triclosan tolerant (MIC≥0.25).**
(DOC)

Table S2 **Antibiotic susceptibility, of the 64** ***S. epidiermidis*** **isolated from blood in 2010-11, given for the triclosan tolerant isolates (MIC≥0.25 mg/l, n = 8) and for the triclosan susceptible (n = 56).**
(DOC)
