# Supplementary material for: Staphylococcus epidermidis Isolated in 1965 Are More Susceptible to Triclosan than Current Isolates
Source: PLoS One. 2013 Apr 16;8(4):e62197. doi: 10.1371/journal.pone.0062197 (PMC3628582; doi:10.1371/journal.pone.0062197)
Supplement: Table S1 — Multilocus sequence typing (MLST) was performed for 15 S. epidermidis isolated from blood in 2010-11, 7 triclosan susceptible (MIC<0.25 mg/l) and 8 triclosan tolerant (MIC≥0.25). (DOC) [file pone.0062197.s001.doc]

**Table S1.** Multilocus sequence typing (MLST) was performed for 15 *S. epidermidis* isolated from blood in 2010-11, 7 triclosan susceptible (MIC<0.25 mg/l) and 8 triclosan tolerant (MIC≥0.25).

| **Isolate** | **Year** | **TCS** | **ST** | **arcC** | **aroE** | **gtr** | **mutS** | **pyr** | **tpi** | **yqiL** |
| --- | --- | --- | --- | --- | --- | --- | --- | --- | --- | --- |
| BD-09 | 2010 | <0.25 | 5 | 1 | 1 | 1 | 2 | 2 | 1 | 1 |
| BD-10 | 2010 | <0.25 | 32 | 1 | 1 | 7 | 1 | 3 | 5 | 14 |
| BD-26 | 2011 | <0.25 | ND | 12 | ? | 5 | 5 | 3 | new? | 4 |
| BD-44 | 2011 | <0.25 | 5 | 1 | 1 | 1 | 2 | 2 | 1 | 1 |
| BD-50 | 2011 | <0.25 | 73 | 1 | 5 | 2 | 6 | 2 | 1 | 6 |
| BD-62 | 2011 | <0.25 | 327 | 1 | 1 | 2 | 1 | 4 | 1 | 1 |
| Van-1 | 2011 | <0.25 | 2 | 7 | 1 | 2 | 2 | 4 | 1 | 1 |
| BD-06 | 2010 | ≥0.25 | 88 | 1 | 1 | 2 | 1 | 2 | 1 | 7 |
| BD-12 | 2011 | ≥0.25 | 88 | 1 | 1 | 2 | 1 | 2 | 1 | 7 |
| BD-18 | 2011 | ≥0.25 | 73 | 1 | 5 | 2 | 6 | 2 | 1 | 6 |
| BD-19 | 2011 | ≥0.25 | 88 | 1 | 1 | 2 | 1 | 2 | 1 | 7 |
| BD-24 | 2011 | ≥0.25 | ND | 3 | ? | 16 | 5 | 11 | 4 | 4 |
| BD-32 | 2011 | ≥0.25 | 2 | 7 | 1 | 2 | 2 | 4 | 1 | 1 |
| BD-38 | 2011 | ≥0.25 | new | 7 | 1 | 2 | 2 | 4 | new | 1 |
| BD-53 | 2011 | ≥0.25 | 5 | 1 | 1 | 1 | 2 | 2 | 1 | 1 |

TCS: triclosan minimum inhibitory concentrations in mg/l. ND=not determined.
