# Supplementary material for: Staphylococcus epidermidis Isolated in 1965 Are More Susceptible to Triclosan than Current Isolates
Source: PLoS One. 2013 Apr 16;8(4):e62197. doi: 10.1371/journal.pone.0062197 (PMC3628582; doi:10.1371/journal.pone.0062197)
Supplement: Table S2 — Antibiotic susceptibility, of the 64 S. epidiermidis isolated from blood in 2010-11, given for the triclosan tolerant isolates (MIC≥0.25 mg/l, n = 8) and for the triclosan susceptible (n = 56). (DOC) [file pone.0062197.s002.doc]

**Table S2.**  Antibiotic susceptibility, of the 64 *S. epidiermidis* isolated from blood in 2010-11, given for the triclosan tolerant isolates (MIC≥0.25mg/l, n=8) and for the triclosan susceptible (n=56).

|  | **MIC<0.25 mg/l** | **MIC≥0.25 mg/l** | **Fishers exact test** |
| --- | --- | --- | --- |
| **Penicillin** | 98% | 100% | Not significant |
| **Cefoxitin** | 61% | 75% | Not significant |
| **Fucidic acid** | 63% | 100% | Not significant |
| **Gentamicin** | 43% | 13% | Not significant |
| **Erythromycin** | 46% | 25% | Not significant |
| Clindamycin | 32% | 13% | Not significant |
| **Rifampicin** | 2% | 0% | Not significant |
| **Linezolid** | 0% | 0% | Not significant |
| **Norfloxacin** | 48% | 25% | Not significant |

Antibiotic susceptibility is given as percent resistant isolates for each antibiotic in the two groups
